# Matching clinical and genetic diagnoses in autosomal dominant polycystic kidney disease reveals novel phenocopies and potential candidate genes

**DOI:** 10.1038/s41436-020-0816-3

**Published:** 2020-05-13

**Authors:** Ria Schönauer, Sebastian Baatz, Melanie Nemitz-Kliemchen, Valeska Frank, Friederike Petzold, Sebastian Sewerin, Bernt Popp, Johannes Münch, Steffen Neuber, Carsten Bergmann, Jan Halbritter

**Affiliations:** 1 Department of Internal Medicine, Division of Nephrology, University Hospital Leipzig, Leipzig, Germany; 2 Institute of Human Genetics, Bioscientia, Ingelheim, Germany; 3 Medizinische Genetik Mainz, Limbach Genetics, Mainz, Germany; 4 Institute of Human Genetics, University of Leipzig, Leipzig, Germany; 5 Department of Medicine, Division of Nephrology, University Hospital Freiburg, Freiburg, Germany

**Keywords:** ADPKD, *FLCN*, *ALG9*, Birt–Hogg–Dubé syndrome, *PKHD1*

## Abstract

**Purpose:**

Autosomal dominant polycystic kidney disease (ADPKD) represents the most common hereditary nephropathy. Despite growing evidence for genetic heterogeneity, ADPKD diagnosis is still primarily based upon clinical imaging criteria established before discovery of additional PKD genes. This study aimed at assessing the diagnostic value of genetic verification in clinical ADPKD.

**Methods:**

In this prospective, diagnostic trial, 100 families with clinically diagnosed ADPKD were analyzed by PKD gene panel and multiplex ligation-dependent probe amplification (MLPA); exome sequencing (ES) was performed in panel/MLPA-negative families.

**Results:**

Diagnostic *PKD1/2* variants were identified in 81 families (81%), 70 of which in *PKD1* and 11 in *PKD2*. *PKD1* variants of unknown significance were detected in another 9 families (9%). Renal survival was significantly worse upon *PKD1* truncation versus nontruncation and *PKD2* alteration. Ten percent of the cohort were *PKD1*/*2-*negative, revealing alternative genetic diagnoses such as autosomal recessive PKD, Birt–Hogg–Dubé syndrome, and *ALG9*-associated PKD. In addition, among unsolved cases, ES yielded potential novel PKD candidates.

**Conclusion:**

By illustrating vast genetic heterogeneity, this study demonstrates the value of genetic testing in a real-world PKD cohort by diagnostic verification, falsification, and disease prediction. In the era of specific treatment for fast progressive ADPKD, genetic confirmation should form the basis of personalized patient care.

## INTRODUCTION

With a prevalence of 1:1000, autosomal dominant polycystic kidney disease (ADPKD) is the most common hereditary nephropathy accounting for about 10% of end-stage renal disease (ESRD).^1^ While ADPKD is a systemic condition, its hallmark is adult-onset bilateral cystic kidney enlargement leading to inflammation, hypertension, and progressive impairment of renal function. The main extrarenal manifestation is polycystic liver disease (PCLD) that is present in up to 94% of patients after the age of 35.^2^

Although the two main causative genes, *PKD1* (encoding polycystin 1; PC1) and *PKD2* (encoding polycystin 2; PC2), have long been identified, the mechanism of disease initiation remains incompletely understood. While pathogenic variants in *PKD1* account for 75–80% of cases, 10–15% of patients are found to harbor pathogenic variants in *PKD2*.^3^ Patients with *PKD1-*associated disease develop ESRD on average 20 years earlier than those with pathogenic *PKD2* germline variants (58 vs. 80 years).^4^ Multiple lines of evidence indicate that disease progression mainly depends on the functional level of PC1. Interestingly, about 10% of patients are reported to carry no pathogenic variant in *PKD1*/*2*, suggesting genetic heterogeneity.^3^ More recently, pathogenic variants in a third gene, *GANAB*,^5^ were described to result in a milder renal phenotype with predominating cystic liver disease. Additionally, PKD phenocopies due to genetic variants in a number of further genes such as *HNF1β*, *PKHD1*, *DNAJB11*, or *TSC1*/*2* have been reported.^6,7^

In clinical practice, diagnosis of ADPKD is based upon imaging criteria without genetic confirmation. Diagnostic criteria comprise the number of renal cysts on ultrasound or magnetic resonance imaging (MRI) along with family history and claim to provide a sensitivity and specificity of almost 100%.^8,9^ However, these criteria were introduced well before the discovery of new genetic forms and increased recognition of ADPKD phenocopies (e.g., ADPKD-*GANAB*, atypical ADPKD-*DNAJB11*). To date, the ability of these criteria to distinguish ADPKD from its phenocopies has not been systematically evaluated. In the era of specific drug treatment (e.g., tolvaptan), detecting fast disease progression at early stages has become crucial for the selection of patients who will benefit the most from new therapies. Apart from imaging (total kidney volume, TKV^10^), risk prediction can be improved through genetic information, as evidenced by the Predicting Renal Outcomes in ADPKD (PROPKD) score.^4^

In this single-center study, we aimed to assess the diagnostic accuracy in a cohort of clinically diagnosed ADPKD patients by means of extended genetic diagnostics based on copy-number variation analysis (multiplex ligation-dependent probe amplification [MLPA]), targeted (PKD gene panel), and exome sequencing (ES) techniques.

## MATERIALS AND METHODS

All patients clinically diagnosed as ADPKD and presenting at the University of Leipzig Medical Center (Germany) for medical advice between November 2016 and November 2018 were enrolled consecutively. Study participants were recruited from the following outpatient and inpatient sources on the basis of a clinical ADPKD diagnosis in their health record: nephrogenetics outpatients’ clinic, transplant outpatients’ clinic, dialysis unit, nephrology ward, and surgical transplant ward. Written informed consent was obtained from all study participants (Institutional Review Board, Leipzig University/Institute of Human Genetics Bioscientia). ADPKD diagnosis was based on medical and family history as well as on imaging data obtained by renal ultrasound, MRI and/or computed tomography according to previously defined criteria.^8,9^ Calculation of height-adjusted total kidney volume (htTKV) was performed uniformly for all patients with available imaging data by use of the Ellipsoid equation (https://mayo.edu/research/documents/pkd-center-adpkd-classification/doc-20094754). In this way, 122 patients from 100 families of mainly Caucasian decent (German *n* = 92, Italian *n* = 2, Syrian *n* = 2, Afghan *n* = 1, French *n* = 1, UK *n* = 1, US *n* = 1) were screened for pathogenic variants in *PKD1*, *PKD2*, *GANAB*, *PKHD1*, and *HNF1B* and a number of other genes that may phenocopy PKD by a next-generation sequencing (NGS) based gene panel and consecutive MLPA. Genomic DNA was fragmented, coding exons of *PKD1*, *PKD2*, *GANAB*, *PKHD1*, and *HNF1B* as well as exon–intron boundaries were enriched (Roche/NimbleGen sequence capture), amplified, and sequenced on an Illumina platform. NGS data were analyzed using JSI^®^ Medical Systems. Insufficiently covered regions were amplified and subjected to Sanger sequencing. MLPA of *PKD1*, *PKD2*, *PKHD1*, and *HNF1B* was performed using the SALSA kits P351, P352, P341, P343, and P241. For ES analysis, genomic DNA was fragmented, and exonic regions plus exon–intron boundaries were enriched (Roche NimbleGen; SeqCap MedExome Library), amplified, and sequenced on an Illumina platform. NGS data were aligned to the hg19 genome assembly. For analysis of renal survival, defined by absence of end-stage renal disease and/or absence of renal replacement therapy, characterized by an eGFR greater than 15 ml/min/1.73m^2^ Chronic Kidney Disease Epidemiology Collaboration (CKD-EPI),^11^ we used log-rank statistical testing. Nonsense, frameshift, splice site variants, and (large) deletions/insertions were labeled as truncating, whereas missense variants and small in-frame deletions/insertions (delins) were categorized as nontruncating in the context of renal survival analysis. Identified SNVs and delins were filtered for variants with a minor allele frequency (MAF) of <1% in gnomAD and known artifacts as well as variants in highly homologous regions were removed. Variant classification was based on published diagnostic criteria of the American College of Medical Genetics and Genomics (ACMG)^12^: class 1—benign; class 2—likely benign, class 3—variant of unknown significance (VUS), class 4—likely pathogenic, class 5—pathogenic.^12^ Hereafter, class 4 and 5 variants are termed diagnostic variants.

## RESULTS

### Overall characteristics of clinically diagnosed ADPKD cohort

The study cohort comprised 122 clinically diagnosed ADPKD patients, 51 females and 71 males, from 100 families of mainly Caucasian decent (97/100; 97%) (Table S1). A positive family history was present in 94% (61/69) of patients. In 31 cases, data on family history were inconclusive. Mean age was 56.7 years, mean body mass index (BMI) 26.0 kg/m^2^, and mean eGFR 34.3 ml/min/1.73m^2^ (CKD-EPI) including all CKD stages (G1–G5). However, with 56% (68/122) of the cohort in ESRD, CKD stage G5 predominated (Table S1). As to be expected, polycystic liver disease represented the most frequent extrarenal manifestation (76%; 93/122) (Table S1).

### Genetic analysis of clinically diagnosed ADPKD cohort

With a mean coverage of 441× for the NGS panel and a mean coverage of 129× for ES, sufficient diagnostic sequencing depth was reached for both methods.

**Fig. 1:**
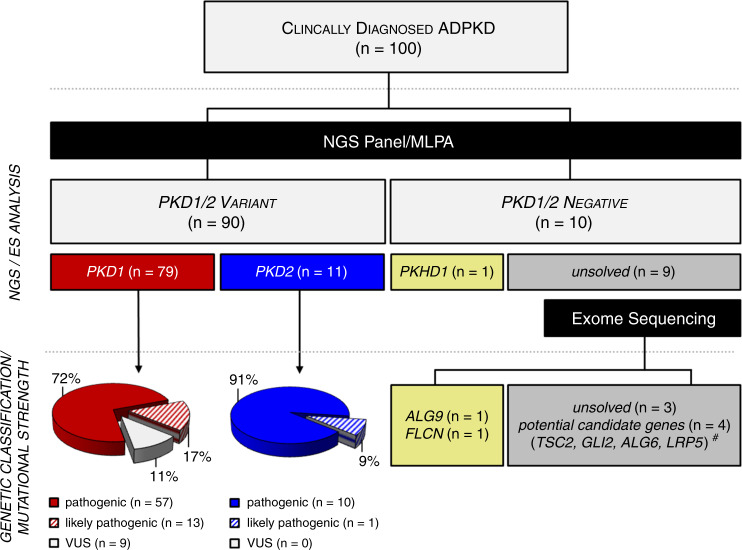
Study design and outcomes. 100 clinically diagnosed autosomal dominant polycystic kidney disease (ADPKD) families were genetically analyzed by next-generation sequencing (NGS)-based customized PKD gene panel and multiplex ligation-dependent probe amplification (MLPA). As a result, class 3–5 *PKD1/2* variants were identified in 90 families (79 *PKD1—*red, although 9 of these were formally *PKD1*-VUS, 11 *PKD2—*blue). In the remaining 10 families without identification of class 3–5 *PKD1*/*2* variants (*PKD1/2-*negative), panel diagnostics yielded two known heterozygous *PKHD1* variants (yellow), indicating autosomal recessive PKD (ARPKD) in one family. In another two families, exome sequencing (ES) identified a pathogenic *FLCN* variant (yellow) and a pathogenic *ALG9* variant (yellow) respectively. Among seven families who remained genetically unsolved (gray box), we identified four potential candidate gene variants in *ALG6*, *GLI2*, *LRP5*, and *TSC2* (#) (Fig. S2). *VUS* variant of unknown significance.

By means of panel diagnostics and MLPA, we were able to confirm ADPKD on a genetic basis in 81 families (81%) by detection of 70 diagnostic *PKD1* and 11 diagnostic *PKD2* variants. Additionally, 9 families (9%) harbored likely causative but formally nondiagnostic variants of unknown significance (VUS class 3) in *PKD1*. In the remaining 10% (10/100) of families, no class 3–5 ACMG variants (VUS, likely pathogenic, pathogenic) were identified in *PKD1* or *PKD2*, hence termed “*PKD1*/*2*-negative” (Fig. 1). However, in one of these families, NGS panel diagnostics yielded an alternative PKD diagnosis in the form of autosomal recessive disease (biallelic compound heterozygous *PKHD1* variants), and in the 9 unresolved families (9%), consecutive ES resulted in alternative genetic diagnoses in another two families by identification of pathogenic variants in *ALG9* and *FLCN* (Fig. 1).

Altogether, 95 different most likely disease-associated variants were identified in our cohort: 82 in *PKD1*, 9 in *PKD2*, 2 in *PKHD1*, 1 in *ALG9*, and 1 in *FLCN*. Among these, there was a total of 40 (42%; 40/95) novel gene variants that have not been previously associated with PKD in the literature (Table S2).

**Fig. 2:**
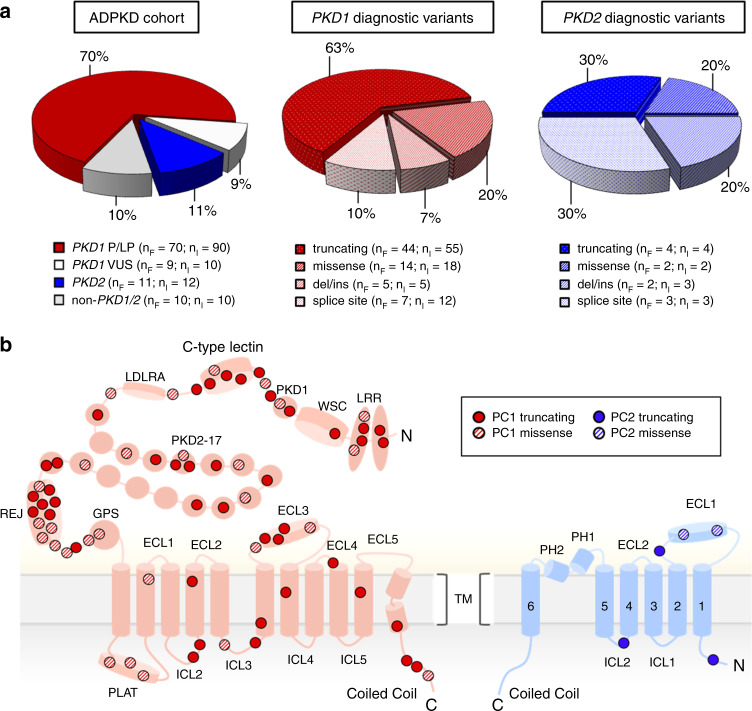
Genetic findings and polycystin (PC)1/2 two-dimensional domain structure. (**a**) Pie charts indicating distribution of genetic findings in total autosomal dominant polycystic kidney disease (ADPKD) cohort, among *PKD1* carriers, and among *PKD2* carriers. *LP* likely pathogenic (diagnostic variant), *P* pathogenic (diagnostic variant). *n*_F_ = number of families; *n*_I_ = number of individuals. (**b**) Illustration of the two-dimensional domain structure of PC1 (red) and PC2 (blue), highlighting genetic findings of this study. Of note, known critical PC1 domains, such as REJ/GPS and C-type lectin, show clustering of pathogenic variants. *ECL* extracellular loop, *ICL* intracellular loop, *LRR* leucin-rich repeats, *PH* pore helix, *WSC* cell wall integrity and stress response component.

With regards to genic and allelic composition, 70% of all individuals were classified as *PKD1* and 11% as *PKD2-*associated disease (Fig. 2a), under strict nonconsideration of nine *PKD1*-VUS cases. While in *PKD1*, 63% of diagnostic changes represented truncating variants, in *PKD2* genetic findings were almost equally distributed between truncating, missense, splice site, and delins variants (Fig. 2a). By schematic representation of affected amino acid residues, we did not observe a mutational hotspot, but instead identified variants scattered across PC1–PC2 protein structures, though two functionally important PC1 regions surrounding the REJ/GPS and lectin domains appeared as clusters in our cohort (Fig. 2b).

In 10 individuals (8%; 10/122), we detected more than a single heterozygous *PKD1* class 3–5 variant (Table S3). In all of these cases, the second allele constituted a VUS in addition to a (likely) pathogenic variant. In one patient (ID109.2) with postnatal kidney cysts and early-onset hypertension, a likely pathogenic *PKD1* variant was identified in *trans* with a class 3 variant possibly explaining congenital disease manifestation by way of disease modification (Table S3). Intrafamilial variability was demonstrated by individuals 12.1/12.4 and 37.1/37.2, respectively, showing strikingly discrepant renal survival despite identical *PKD1* findings (Table S3). Conversely, ID31.1 with most progressive renal disease (ESRD at age 39) was found to harbor as many as three different *PKD1* variants (1× pathogenic, 2× VUS) (Table S3).

### Phenotype–genotype correlations in *PKD1* vs. *PKD2* vs. *PKD1*/*2*-negative cases

**Fig. 3:**
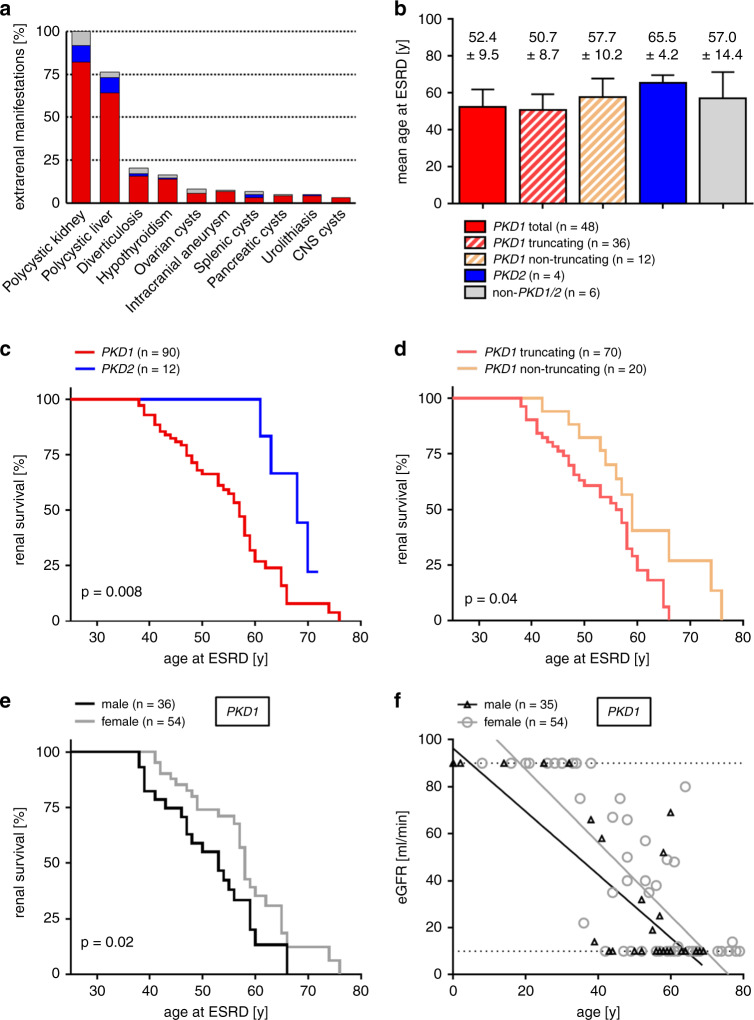
Phenotype–genotype correlations. (**a**) Spectrum of extrarenal manifestations according to genotypic groups: *PKD1* total—red; *PKD2* —blue, *PKD1*/*2*-negative—gray. (**b**) Mean age at end-stage renal disease (ESRD) including standard deviation in years according to different genotypes. (**c**) Kaplan–Meier curve, showing significant difference in renal survival between *PKD1* (red; median = 57 years) and *PKD2* carriers (blue; median = 68 years) and between carriers of a *PKD1*-truncating (red; median = 56 years) and a *PKD1*-nontruncating variant (orange; median = 59 years) (**d**). (**e**) Kaplan–Meier curve, showing significant difference in renal survival between male (median = 53 years) and female (median = 58 years) autosomal dominant polycystic kidney disease (ADPKD) patients, and differences in estimated glomerular filtration rate (eGFR) (CKD-EPI) at age according to sex (**f**). Statistic calculation was done by use of log-rank test (GraphPad; Prism). Renal survival is defined as absence of ESRD/renal replacement therapy. *CNS* central nervous system.

After stratifying groups according to molecular genetic diagnoses (*PKD1*, *PKD2*, and *PKD1/2*-negative), we analyzed for phenotypic differences. While the three groups showed comparable BMI, their mean eGFR (33 vs. 45 vs. 32 ml/min/1.73m^2^, CKD-EPI) and mean htTKV (1399 vs. 1075 vs. 657 ml/m) differed significantly between *PKD1* and *PKD2* carriers and *PKD1/2*-negative cases (Table S1). Cystic liver involvement was more frequent in *PKD1* and *PKD2* carriers (78% and 92%) than in *PKD1/2*-negative subjects (40%) (Table S1). Other extrarenal manifestations did not vary between genetically defined subgroups (Fig. 3a). However, *PKD1* carriers had significantly poorer renal prognosis than those carrying *PKD2* variants, with mean age at ESRD onset of 52.4 versus 65.5 years (Fig. 3b, c). Furthermore, in carriers of *PKD1* truncating variants, ESRD onset was seven years earlier when compared with carriers of *PKD1* nontruncating variants and almost 15 years earlier when compared with *PKD2* carriers (Fig. 3b–d). Mean age at ESRD onset in the *PKD1*/*2*-negative group (57 years) was in between both groups (Fig. 3b). Lastly, among *PKD1* carriers, male sex was associated with significantly poorer renal prognosis, as has been reported previously^4^ (Fig. 3e, f).

### Identification of ADPKD phenocopies in patients without diagnostic *PKD1*/*2* variants

**Fig. 4:**
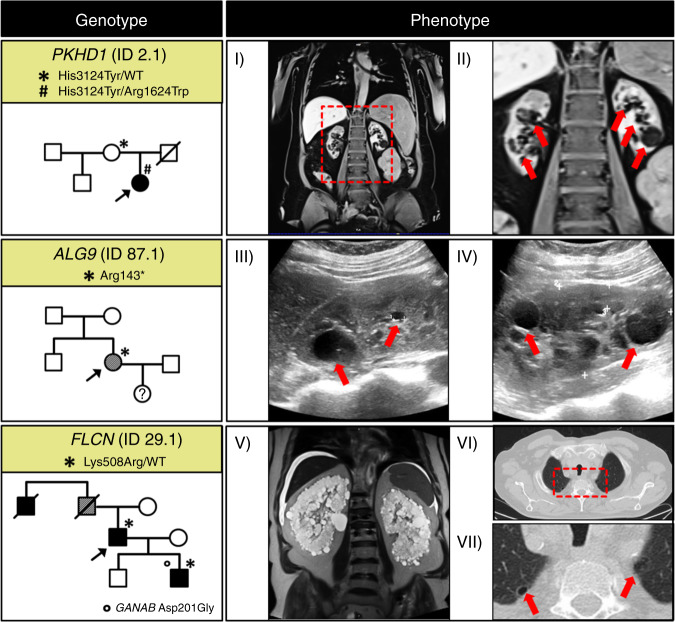
Identified autosomal dominant polycystic kidney disease (ADPKD) phenocopies in three families. Genotypes and phenotypes (including pedigrees) of families with identified APDKD phenocopies: upper panel (ID2.1): ARPKD (autosomal recessive polycystic kidney disease) due to pathogenic compound heterozygous *PKHD1* variants (p.His3124Tyr, transmitted maternally/p.Arg1624Trp). Bilateral kidney cysts are indicated by red arrows. (I–II) Abdominal magnetic resonance imaging (MRI) shows bilateral polycystic kidney disease (red arrows) at the age of 45; 15 years after onset of end-stage renal disease (ESRD). Of note, childhood imaging was not available when bilateral kidney enlargement was reported. Middle panel (ID87.1): *ALG9*-associated PKD due to a heterozygous *ALG9* truncating variant (p.Arg143*). (III–IV) Renal ultrasound shows multiple kidney cysts (bilateral) at the age of 31. Lower panel (ID29.1): Birt–Hogg–Dubé syndrome (BHDS) due to a pathogenic heterozygous *FLCN* variant (p.Lys508Arg). Of note, the son of the index patient who was found to harbor the *FLCN* missense variant in addition to a *GANAB*-VUS (p.Asp201Gly without entry in gnomAD) was also diagnosed with ESRD at age 30 due to bilateral PKD. (V) Abdominal MRI showing bilateral polycystic kidney disease with significant organ enlargement at the age of 65. (VI–VII) Computed tomography of the chest shows minimal signs of cystic lung disease (red arrows). Index patients are denoted by black arrows. Patients with ESRD are illustrated by filled black symbols while patients with CKD stage 3–4 are denoted by hatched symbols.

Among the ten patients without any class 3–5 *PKD1*/*2* findings, we identified three alternative molecular diagnoses: ARPKD (MIM 263200), *ALG9-*associated PKD, and Birt–Hogg–Dubé syndrome (BHDS, MIM 135150) (Table 1, Fig. 4).

**Table 1:** Pathogenic variants identified in three *PKD1/2-*negative patients.

ID	Sex	Age (years)	eGFR (ml/min/1.73m^2^)	Age at ESRD (years)	Gene	Variant (c.) zygosity	Variant (p.)	ACMG gnomAD_AF_	Ref.	Extrarenal phenotype
2.1	F	47	<10	30	*PKHD1*	c.4870C>T het c.9370C>T het	p.Arg1624Trp p.His3124Tyr	P 0.02% P none	HGMD HGMD	No liver cysts Ovarian cysts HPT
87.1	F	31	>90	–	*ALG9*	c.427C>T het	p.Arg143*	P 0.0016%	HGMD	No liver cysts
29.1	M	66	<10	56	*FLCN*	c.1523A>G het	p.Lys508Arg	P 0.04%	HGMD	No liver cysts DV

Given gnomAD allele frequencies (gnomAD_AF_) refer to the corresponding ancestry of the index patient, in these cases European non-Finnish. *None* indicates no entry in gnomAD.

*ACMG* American College of Medical Genetics and Genomics, *DV* diverticulosis, *eGFR* estimated glomerular filtration rate (CKD-EPI; ml/min/1.73m^2^), *ESRD* end-stage renal disease, *F* female, *HGMD* Human Gene Mutation Database (version 2019.4), *HPT* hypothyroidism, *M* male, *P* pathogenic.

Of note, 6/10 *PKD1/2*-negative patients had a positive family history and met diagnostic imaging criteria^8,9^ (>2 cysts bilaterally plus positive family history); however, absence of PCLD was a common phenomenon in this group (6/10) (Fig. 4, Table S4, Fig. S1).

In ID2.1, a 47-year-old female with negative family history, bilateral childhood-onset cystic kidneys and ESRD at age 30, two pathogenic, previously reported, compound heterozygous *PKHD1* missense variants were identified (c.4870C>T, p.Arg1624Trp and c.9370C>T, p.His3124Tyr)^13,14^ (Table 1, Fig. 4). Interestingly, this patient did not exhibit any liver abnormalities.

Furthermore, in ID87.1, a 31-year-old female with negative family history, presenting with preserved kidney function and bilateral kidney cysts, we detected a heterozygous *ALG9* truncating variant (c.427C>T, p.Arg143*) (Table 1, Fig. 4). No liver abnormalities were seen on abdominal ultrasound. Interestingly, monoallelic variants in *ALG9* encoding the ER (endoplasmic reticulum) enzyme ɑ-1,2-mannosyltransferase were just recently identified in genetically unresolved, atypical ADPKD, including this very truncating variant, which had been found associated with mild bilateral PKD.^15^

Lastly, in ID29.1, a 66-year-old male with positive family history, ESRD at age 56, and bilateral polycystic kidneys, ES yielded a previously reported heterozygous *FLCN* missense variant (c.1523A>G, p.Lys508Arg)^16^ compatible with BHDS (Table 1, Fig. 4). Upon retrospective phenotyping, discrete manifestations of cystic lung disease but no signs of pneumothorax, renal, dermal, or gastrointestinal malignancies could be detected (Table 1, Fig. 4). Interestingly, this patient reported that his son had been clinically diagnosed with ADPKD and been on hemodialysis since the age of 30. Through gene panel analysis, we also ruled out pathogenic *PKD1*/*2* variants and confirmed the identical *FLCN* missense change (p.Lys508Arg) in the son. In contrast to his father, however, he also showed an additional VUS in *GANAB*/*PKD3* (c.602A>G, p.Asp201Gly, no entry in gnomAD/HGMD) potentially explaining the accelerated course of renal disease (Fig. 4) (ESRD-onset at 30 vs. ESRD-onset at 56 yrs).

In the remaining seven patients with bilateral polycystic kidney disease (Fig. S1), ES did not yield a definite molecular diagnosis. However, by variant data analysis with a manually curated list of PKD candidates we detected a novel, heterozygous *GLI2* variant (c.1600G>A, p.Val534Met) affecting the DNA-binding zinc-finger domain of the transcription factor (ID20.1) (Table S4, Fig. S2). GLI2 acts as an effector of Sonic hedgehog signaling (Shh), a pathway that was previously observed to be activated in human and murine tissues of ADPKD and non-ADPKD cystic kidney disease.^17^ While loss-of-function of *GLI2* is associated with hypopituitarism and holoprosencephaly (MIM 615849/610829) it was also found to alleviate cystic kidney disease in a murine PKD model, suggesting that gain of function may exert an opposite effect.^18^ ID20.1 reported a negative family history and presented with bilateral PKD without liver cysts and ESRD at the age of 64. Of note, cerebral abnormalities and overt alterations of hypothalamic–pituitary hormone axes were absent, making loss-of-function unlikely for this specific variant. Additionally, microcystic lung fibrosis was diagnosed upon thoracic computed tomography (Fig. S1), a finding potentially in line with profibrotic effects of increased GLI2 transcriptional activity^19^ and a pathogenic role of Shh activation in lung fibrosis (Fig. S2).^20^

In another female *PKD1*/*2*-negative patient (ID97.1) with ESRD at age 68 and liver transplantation for severe PCLD, we identified two rare missense variants in *ALG6* (c.1465T>G, p.Phe489Val and c.1442A>G, p.Asn481Ser) encoding the ER enzyme ɑ-1,3-glucosyltransferase, an interactor of ALG8, which was previously found to be associated with a mild and atypical form of PKD and PCLD^21^ (Table S4, Fig. S2).

Furthermore, several VUS in known renal disease genes were analyzed by means of retrospective phenotyping or upon complementary DNA (cDNA)-based splice site analysis (*GANAB*: c.2319G>A, p.Ala773=, *PARN*: c.1405+3A>G in ID52.1*, SEC63*: c.1936–8->TTT in ID20.1 and ID97.1), disproving pathogenicity (Table S4, Fig. S3). Three pathogenic or likely pathogenic variants observed in *DCHS1* (MIM 607829; ID87.1) and *COL4A3* (MIM 104200; ID52.1) either did not match the phenotype or failed to segregate. Nonsegregation also occurred in ID108.1, a young female with positive family history and several bilateral kidney cysts at the age of 20 (Table S4, Fig. S1). Although her maternal grandmother and mother were found to carry a novel, likely pathogenic *PKD1* in-frame deletion (c.4319_4321delCCT, p.Ser1440del), she turned out *PKD1/2*-negative, carrying a novel, well-conserved *LRP5* missense variant (c.1555T>G, p.Trp519Gly) that was inherited from her unaffected father. The affected amino acid residue lies within the second WD40-repeat of the Wnt coreceptor LRP5 (low-density lipoprotein receptor-related protein 5), a domain that was recently associated with cystic kidney and liver disease^22^ (MIM 617875) by identification of a neighboring tryptophan change (p.Trp560Cys), potentially indicating incomplete penetrance in this family (Table S4, Fig. S2). Lastly, in ID35.1, a 64-year-old male with positive family history, bilateral oligocystic kidney disease, a pancreatic cyst formation, and moderately impaired kidney function (CKD stage 3b with an eGFR at 41 ml/min), we detected a novel heterozygous *TSC2* missense variant (c.2140C>T, p.Pro714Ser) affecting the well-conserved tuberin domain, a region in which missense variants have commonly been associated with tuberous sclerosis complex (TSC, MIM 613254). Although of interest, retrospective phenotyping did not reveal additional diagnostic signs or symptoms of TSC, such as cerebral or skin manifestation, and additional family members were unavailable for segregation analysis. Therefore, we presently classified this variant as nondiagnostic VUS (Table S4, Fig. S1).

## DISCUSSION

With 79% *PKD1* carriers (including 9% *PKD1*-VUS carriers) and 11% *PKD2* carriers, the results of our investigation reflect the genetic distribution found in larger ADPKD cohort studies.^3,23^ Importantly, genotypic differences in renal survival confirmed previous reports^3,4,24^ although *PKD2* patients appeared more severely affected, underlined by the high proportion of polycystic liver disease among *PKD2* carriers in our cohort (92%). While previous studies yielded a mean age at ESRD onset of 75–80 years for *PKD2*-associated disease, patients in our study developed ESRD about 10 years earlier, yet significantly later than *PKD1* carriers. The more severe clinical presentation of *PKD2* carriers in our study may be explained by selection bias toward ESRD patients and a relatively small total number of *PKD2* patients.

Interestingly, despite this moderate-sized cohort and a potential selection bias toward increased severity in *PKD2*, we were able to demonstrate significant prognostic value of PKD genotyping for renal disease prediction. Furthermore, we confirmed the tendency to faster disease progression among males.^3,4,24^ In ten individuals, we identified more than one *PKD1* variant. These patients displayed a combination of a pathogenic variant and a VUS, indicating potential genetic modification within these families. In accordance with the concept of PC1 dosage, one of our patients carrying a likely pathogenic *PKD1* variant and a *PKD1*-VUS in *trans* showed a severe, congenital phenotype (ID109.2). Another severely affected individual (ID31.1) with ESRD at 39 years of age was even found to harbor three different *PKD1* variants (1× pathogenic and 2× VUS). While segregation data were lacking, it can only be suspected that two of these changes reside on different parental alleles.

A major obstacle to the genetic analysis in ADPKD is a reduced sensitivity of noncustomized NGS-based methods in reliably detecting *PKD1* variants due to its pseudogene regions on chromosome 16.^25^ Notably, ES was recently shown to miss a critical amount of *PKD1* variants in the duplicated regions of exons 1–32, which make up most of the gene.^25^ To meet this challenge, our PKD gene panel was specifically designed to enrich these duplicated regions of *PKD1* to guarantee a sufficient sequencing depth and a low rate of false positives and false negatives.^26^

With 10%, the amount of *PKD1/2*-negative patients resembles the rate in other, ethnically divergent cohorts from India (15%), Italy (20%), France (10%), China (14%), and Japan (17%).^3,23,27–29^ Taken together, these data indicate inaccuracy of clinical ADPKD diagnoses in at least 10% of cases and about twice as much if VUS carriers are strictly counted as nondiagnostic (additional 9% in our cohort).

Due to recent progress in gene identification, a growing number of conditions were characterized that present with clinical findings similar to ADPKD but different underlying genetic causes and mechanisms. Examples of these ADPKD phenocopies are *DNAJB11-*associated polycystic liver and kidney disease (MIM 600666/MIM 618061), autosomal recessive polycystic kidney disease (ARPKD, MIM 263200), TSC (MIM 191100/613254), Von Hippel–Lindau (VHL) disease (MIM 193300), *LRP5* (MIM 617875),^22^ and *HNF1B-*associated cystic kidney disease (MIM 137920). Unlike previous studies, *PKD1/2*-negative cases prompted us to conduct further genetic analyses to unravel the underlying cause. Through extended genetic diagnostics, we were able to establish an alternative PKD diagnosis in 3 of 10 families (30%). Indeed, the finding of biallelic *PKHD1* missense variants emphasized the role of ARPKD as differential diagnosis in adult PKD with a negative family history. However, we did not detect pathogenic variants in *GANAB*/*PKD3*, *DNAJB11*, or *HNF1B*. Instead, we identified pathogenic variants in *FLCN* and *ALG9*, extending the spectrum of ADPKD phenocopies to Birt–Hogg–Dubé syndrome (BHDS) and *ALG9-*related PKD.^6^ By this, we corroborate the recent observation that heterozygous *ALG9* loss-of-function variants,^15^ similar to *ALG8*,^21^ contribute to polycystic kidney and liver disease, in line with cumulative evidence of a continuous disease spectrum between both cystic disorders.^21,30^ Our finding of two missense variants in *ALG6*, encoding the enzymatic partner of ALG8 during the first step of ER-based N-glycosylation, may suggest a similar contribution, although that is hypothetical at this stage.

In BHDS, its variable clinical presentation and the lack of typical pulmonary manifestations in many patients often lead to a delayed diagnosis.^31^ Interestingly, the *FLCN* missense variant (p.Lys508Arg) identified in our patient has been extensively investigated recently. Whereas normal tumor suppressor activity was shown in vitro,^32^ transgenic expression of *FLCN* Lys508Arg in heterozygous *Flcn* knockout mice was associated with bilateral cystic kidney disease, suggesting a dominant-negative effect.^33^ Analogously, we did not find any signs of malignancies and only minimal cystic lung manifestation in our patient, but severe bilateral cystic kidney disease with dominant transmission (Fig. 4). Hence, BHDS can well mimic the clinical picture of ADPKD, rendering it a novel ADPKD phenocopy. Interestingly, FLCN was recently implicated in regulating mTORC1 via primary cilia, suggesting a ciliary link between PC1 and FLCN.^34^

Accuracy of diagnostic algorithms is critical, not only in the context of clinical trials, but also in clinical routine, when deciding on which patients should receive new targeted therapies. While ADPKD is still considered a clinical diagnosis based on its relatively characteristic phenotype, treatment should be initiated at an early stage, when cyst growth is still moderate and phenotypic presentation is often less striking than in later stages of the disease.^35^ Moreover, recent findings of unbiased genetic trials in CKD cohorts show that an extended clinical spectrum in many hereditary kidney diseases is to be expected;^36^ the most prominent example being genetic focal segmental glomerulosclerosis (FSGS) due to mutated *COL4A3*–*5* genes, a group of genes that had previously been associated with Alport syndrome only.^37^ Genetic and clinical heterogeneity of PKD is becoming increasingly evident, especially when taking into account recent findings of ARPKD phenocopies, such as *DZIP1L* and *ANKS6*.^38,39^ As imaging criteria for the diagnosis of ADPKD were developed before the identification of additional dominant PKD genes such as *GANAB*, *ALG8*, *ALG9*, *LRP5*, and *DNAJB11*, they do not reliably apply to these disorders and may require reevaluation.

This study has several limitations: first, the cohort was only moderate in size, ethnically homogeneous, and derived from a single center, therefore limiting generalizability. Second, although applying extensive genetic diagnostics, we cannot strictly exclude having missed pathogenic *PKD1*/*2* variants in deep intronic and/or regulatory regions. Furthermore, cases of genetic mosaicism, although being investigated, could have been overlooked since blood cell–derived DNA only and no DNA from other tissues was analyzed.

In conclusion, our study emphasizes the importance of genetic verification in clinically suspected ADPKD for two main reasons. First, it allows for prediction of disease progression in conjunction with clinical parameters (sex, hypertension, urological event), as demonstrated by the PROPKD score.^4^ Second, genetic analysis enables detection of ADPKD phenocopies and excludes *PKD1*/*2*-negative patients from potentially harmful treatment, given that studies on tolvaptan in ADPKD phenocopies are lacking. On the contrary, patients suffering from conditions such as TSC may need mTOR inhibitor treatment,^40^ which conversely was not shown to be beneficial in ADPKD.^41^ As TSC and BHDS represent tumor syndromes, disease recognition is crucial for affected families, prompting intrafamilial screening for malignancies and leading to differential transplant management with mTOR inhibitors as part of immunosuppressive regimens for those with ESRD.^42^

For these reasons, we propose genetic confirmation to be pursued before initiation of targeted therapies. In ADPKD as well as in other heritable conditions, it is hoped that the armamentarium of specific drugs will eventually differentiate with identified genotypes (e.g., trafficking defects versus nontrafficking defects). This study illustrates the genetic heterogeneity of PKD, leading us to conclude that individualized treatment options require the most accurate diagnostics based on clinical prediagnosis and subsequent genetic verification.

## Supplementary information

Supplementary Information
