# Supplementary material for: Matching clinical and genetic diagnoses in autosomal dominant polycystic kidney disease reveals novel phenocopies and potential candidate genes
Source: Genet Med. 2020 May 13;22(8):1374–83. doi: 10.1038/s41436-020-0816-3 (PMC7394878; doi:10.1038/s41436-020-0816-3)
Supplement: Supplementary file 1 — Supplementary Information [file 41436_2020_816_MOESM1_ESM.docx]

**SUPPLEMENTAL MATERIAL**

**TABLE S1 – Study cohort characteristics of 122 individuals with clinically diagnosed ADPKD**

|  | ***PKD1*-carriers**  **[n=100/122 (82.0%)]** | ***PKD2*-carriers**  **[n=12/122 (9.8%)]** | ***PKD1*/*2*-negative**  **[n=10/122 (8.2%)]** | **Total**  **[n=122]** |
| --- | --- | --- | --- | --- |
| **Mean age [y]** | 52.1 | 58.3 | 59.7 | 56.7 |
| **Sex [n]** |  |  |  |  |
| **Male** | 41/122 | 5/122 | 5/122 | 51/122 |
| **Female** | 59/122 | 7/122 | 5/122 | 71/122 |
| **BMI [kg/m²]**  (mean+SD) | 26.0±4.8 | 26.0±3.3 | 26.3±4.8 | 26.0±4.6 |
| **CKD stage*** |  |  |  |  |
| **G1** | 16/100 | 2/12 | 2/10 | 20/122 |
| **G2** | 9/100 | 2/12 | 0/10 | 11/122 |
| **G3** | 12/100 | 4/12 | 2/10 | 18/122 |
| **G4** | 4/100 | 0/12 | 0/10 | 4/122 |
| **G5** | 58/100 | 4/12 | 6/10 | 68/122 |
| **eGFR [ml/min/1.73m^2^]**  (mean+SD) | 33.3±31.8 | 44.7±30.7 | 32.1±33.0 | 34.3±31.7 |
|  |  |  |  |  |
| **htTKV [ml/m]**  (mean+SD) | 1399±1062 | 1075±849 | 657±nd | 1324±1016 |
|  |  |  |  |  |
| **Extra-renal manifestations** | **yes / no / no data** | **yes / no / no data** | **yes / no / no data** | **yes / no / no data** |
| **PCLD** | 78(78%) / 14(14%) / 8(8%) | 11(92%) / 1(8%) / 0(0%) | 4(40%) / 4(40%) / 2(20%) | 93(76%) / 19(16%) / 10(8%) |
| **Diverticulosis** | 19(19%) / 48(48%) / 33(33%) | 2(17%) / 6(50%) / 4(33%) | 4(40%) / 3(30%) / 3(30%) | 25(21%) / 57(48%) / 40(33%) |
| **Ovarian cysts** | 7(7%) / 55(55%) / 38(38%) | 0(0%) / 7(58%) / 5(42%) | 2(20%) / 6(60%) / 2(20%) | 9(7%) / 68(56%) / 45(37%) |
| **ICA** | 8(8%) / 26(26%) / 65(65%) | 0(0%) / 4(33%) / 8(67%) | 1(10%) / 6(60%) / 3(30%) | 9(7%) / 36(30%) / 76(62%) |
| **CNS**  **cysts** | 4(5%) / 30(30%) / 65(65%) | 0(0%) / 4(33%) / 8(67%) | 0(0%) / 7(70%) / 3(30%) | 5(4%) / 41(34%) / 76(62%) |
| **Pancreatic**  **cysts** | 5(5%) / 51(51%) / 44(44%) | 0(0%) / 9(75%) / 3(25%) | 1(10%) / 7(70%) / 2(20%) | 6(5%) / 67(57%) / 49(40%) |
| **Splenic cysts** | 4(4%) / 69(69%) / 27(27%) | 2(17%) / 9(75%) / 1(8%) | 2(20%) / 6(60%) / 2(20%) | 8(7%) / 84(69%) / 30(25%) |
| **Urolithiasis** | 5(5%) / 70(70%) / 25(25%) | 1(8%) / 8(67%) / 3(25%) | 0(0%) / 8(80%) / 2(20%) | 6(5%) / 86(71%) / 30(25%) |
| **Hypothyroidism** | 17(17%) / 60(60%) / 23(23%) | 1(5%) / 8(67%) / 3(25%) | 2(20%) / 6(60%) / 2(20%) | 20(16%) / 74(60%) / 28(23%) |

BMI: body mass index; CKD: chronic kidney disease; CNS: central nervous system; eGFR: estimated glomerular filtration rate (CKD-EPI; ml/min/1.73m^2^); htTKV: height-adjusted total kidney volume; ICA: intracranial aneurysm; m: male; PCLD: polycystic liver disease; SD: standard deviation. *CKD-stages were based on last estimated GFR-value (CKD-EPI) obtained.

**TABLE S2 – Summary of diagnostic variants (class 4-5) identified in this study, including *PKD1*-VUS (class 3)**

| Gene | Exon/Intron | Type | cDNA change | Protein change | Database | ACMG  gnomAD_AF_ | No | PMID |
| --- | --- | --- | --- | --- | --- | --- | --- | --- |
| *PKD1* | Exon 1 | truncating | *c.74delG* | *p.Gly25Alafs*48* | HGMD/PKDB | P  none | 2 | 21115670 |
| *PKD1* | Exon 3 | missense | *c.308T>G* | *p.Ile103Ser* | HGMD | LP  none | 2 | 29801666 |
| *PKD1* | Exon 4 | truncating | *c.404G>A* | *p.Trp135** | HGMD | P  none | 1 | 29633482 |
| *PKD1* | Exon 4 | truncating | *c.412C>T* | *p.Arg138** | HGMD/PKDB | P  none | 1 | 17582161  25525159 |
| *PKD1* | Exon 4 | missense | *c.415T>C* | *p.Trp139Arg* | novel | LP  none | 1 | - |
| *PKD1* | Exon 5 | truncating | *c.540dupT* | *p.Val181Cysfs*6* | HGMD | P  none | 1 | 31027891 |
| *PKD1* | Exon 5 | truncating | *c.896_897delCT* | *p.Pro299Argfs*71* | novel | P  none | 1 | - |
| *PKD1* | Exon 5 | missense | *c.974A>G* | *p.Tyr325Cys* | HGMD/PKDB | LP  none | 2 | 17582161  23431072 |
| *PKD1* | Exon 5 | truncating | *c.1132C>T* | *p.Gln378** | HGMD | P  none | 1 | 23300259 |
| *PKD1* | Exon 5 | missense | *c.1137C>A* | *p.Asn379Lys* | novel | VUS  none | 1 | - |
| *PKD1* | Exon 5 | truncating | *c.1198C>T* | *p.Arg400** | HGMD/PKDB | P  0.0039% | 2 | 11840199  25525159 |
| *PKD1* | Exon 6 | truncating | *c.1347_1357delinsACC* | *p.Ser449Argfs*67* | novel | P  none | 1 | - |
| *PKD1* | Exon 7 | truncating | *c.1391dupT* | *p.Asp465Argfs*54* | novel | P  none | 1 | - |
| *PKD1* | Exon 7 | missense | *c.1396G>A* | *p.Val466Met* | HGMD/PKDB | VUS  none | 1 | 26453610 |
| *PKD1* | Exon 7 | truncating | *c.1584C>A* | *p.Tyr528** | novel | P  none | 1 | - |
| *PKD1* | Exon 8 | truncating | *c.1670delT* | *p.Leu557Argfs*27* | HGMD/PKDB | P  none | 1 | 22508176 |
| *PKD1* | Exon 9 | missense | *c.1831C>T* | *p.Arg611Trp* | HGMD/PKDB | P  none | 1 | 17574468  23431072 |
| *PKD1* | Exon 9 | in-frame del | *c.1832_1833delinsTT* | *p.Arg611delinsLeu* | novel | VUS  none | 1 | - |
| *PKD1* | Exon 11 | missense | *c.2180T>C* | *p.Leu727Pro* | HGMD/PKDB | LP  none | 1 | 17582161  23431072 |
| *PKD1* | Exon 11 | truncating | *c.2329C>T* | *p.Gln777** | HGMD | P  none | 2 | 25333066 |
| *PKD1* | Exon 12 | missense | *c.2965G>A* | *p.Ala989Thr* | novel | VUS  0.0017% | 3 | - |
| *PKD1* | Exon 14 | truncating | *c.3262_3263insT* | *p.Glu1088fs* | novel | P  none | 1 | - |
| *PKD1* | Exon 15 | in-frame deletion | *c.3415_3423delGTGGCCGGC* | *p.Val1139_Gly1141del* | novel | VUS  none | 1 | - |
| *PKD1* | Exon 15 | truncating | *c.3482G>A* | *p.Trp1161** | novel | P  none | 1 | - |
| *PKD1* | Exon 15 | missense | *c.3490G>A* | *p.Gly1164Arg* | HGMD | LP  none | 1 | 27782177 |
| *PKD1* | Exon 15 | truncating | *c.3543T>A* | *p.Tyr1181** | novel | P  none | 1 | - |
| *PKD1* | Exon 15 | truncating | *c.3850delG* | *p.Ala1284Profs*62* | novel | P  none | 1 | - |
| *PKD1* | Exon 15 | missense | *c.3887T>G* | *p.Val1296Gly* | HGMD | VUS  none | 1 | 31027891 |
| *PKD1* | Exon 15 | truncating | *c.4395dupC* | *p.Val1466Argfs*57* | novel | P  none | 2 | - |
| *PKD1* | Exon 15 | missense | *c.4631T>G* | *p.Val1544Gly* | novel | VUS  none | 1 | - |
| *PKD1* | Exon 15 | truncating | *c.4709delC* | *p.Thr1570Serfs*63* | novel | P  none | 1 | - |
| *PKD1* | Exon 15 | truncating | *c.4951C>T* | *p.Gln1651** | HGMD | P  none | 1 | 22508176 |
| *PKD1* | Exon 15 | missense | *c.5953G>A* | *p.Ala1985Thr* | novel | VUS  none | 2 | - |
| *PKD1* | Exon 15 | in-frame deletion | *c.5976_5978delCAC* | *p.Phe1992_Thr1993delinsLeu* | HGMD/PKDB | P  none | 1 | 11115377  19759016 |
| *PKD1* | Exon 15 | truncating | *c.6199C>T* | *p.Gln2067** | HGMD/PKDB | P  none | 1 | 17582161  25525159 |
| *PKD1* | Exon 15 | truncating | *c.6424delC* | *p.Gln2142Argfs*19* | HGMD | P  none | 1 | 31027891 |
| *PKD1* | Exon 15 | missense | *c.6842C>T* | *p.Ser2281Phe* | HGMD | LP  none | 2 | 27782177 |
| *PKD1* | Exon 15 | truncating | *c.6913_6914delCA* | *p.Gln2305Glufs*114* | HGMD/PKDB | P  none | 1 | 12007219 |
| *PKD1* | Exon 18 | truncating | *c.7288C>T* | *p.Arg2430** | HGMD/PKDB | P  none | 1 | 11012875  25525159 |
| *PKD1* | Exon 19 | in-frame deletion | *c.7585_7605del21* | *p.Lys2529_Tyr2535del* | novel | LP  none | 1 | - |
| *PKD1* | Exon 20 | truncating | *c.7861G>T* | *p.Glu2621** | novel | P  none | 1 | - |
| *PKD1* | Exon 21 | truncating | *c.7915C>T* | *p.Arg2639** | HGMD/PKDB | P  none | 1 | 10854095  25525159 |
| *PKD1* | Exon 21 | truncating | *c.7921C>T* | *p.Gln2641** | HGMD/PKDB | P  none | 1 | 26150605 |
| *PKD1* | Exon 21 | truncating | *c.7987C>T* | *p.Gln2663** | HGMD/PKDB | P  none | 2 | 22508176  27782177 |
| *PKD1* | Exon 23 | missense | *c.8293C>T* | *p.Arg2765Cys* | HGMD | VUS  0.86% | 1 | 19165178  24907393 |
| *PKD1* | Exon 23 | missense | *c.8447T>C* | *p.Leu2816Pro* | HGMD/PKDB | P  none | 1 | 11115377  23431072 |
| *PKD1* | Exon 23 | missense | *c.8474T>C* | *p.Leu2825Pro* | HGMD | VUS  none | 1 | 31027891 |
| *PKD1* | Exon 23 | missense | *c.8497C>T* | *p.Pro2833Ser* | HGMD/PKDB | LP  none | 1 | 26139440 |
| *PKD1* | Exon 23 | missense | *c.8611G>A* | *p.Ala2871Thr* | novel | VUS  0.011% | 1 | - |
| *PKD1* | Exon 23 | truncating | *c.8767C>T* | *p.Gln2923** | HGMD | P  none | 1 | 29606500 |
| *PKD1* | Exon 25 | missense | *c.9035C>T* | *p.Thr3012Met* | PKDB | VUS  0.026% | 1 | *Athena Diag.* |
| *PKD1* | Exon 25 | missense | *c.9044G>T* | *p.Cys3015Phe* | novel | LP  none | 1 | - |
| *PKD1* | Exon 27 | missense | *c.9404C>T* | *p.Thr3135Met* | HGMD/PKDB | P  none | 2 | 24611717 |
| *PKD1* | Exon 27 | missense | *c.9499A>T* | *p.Ile3167Phe* | HGMD | VUS  0.21% | 1 | 11967008 |
| *PKD1* | Exon 27 | truncating | *c.9568G>C* | *p.Gly3190Arg* | novel | LP  none | 1 | - |
| *PKD1* | Exon 29 | missense | *c.9898G>A* | *p.Gly3300Arg* | HGMD/PKDB | LP  0.020% | 1 | 23431072 |
| *PKD1* | Exon 35 | truncating | *c.10560dupG* | *p.Pro3521Alafs*106* | novel | P  none | 4 | - |
| *PKD1* | Exon 36 | truncating | *c.10659delG* | *p.Trp3553CysCysfs*32* | novel | P  none | 1 | - |
| *PKD1* | Exon 36 | truncating | *c.10719dupA* | *p.Gly3574Argfs*63* | novel | P  none | 2 | - |
| *PKD1* | Exon 36 | missense | *c. 10820A>T* | *p.Lys3607Met* | novel | VUS  none | 2 | - |
| *PKD1* | Exon 38 | truncating | *c.11033delT* | *p.Met3678Serfs*6* | novel | P  none | 1 | - |
| *PKD1* | Exon 38 | missense | *c.11156G>A* | *p.Arg3719Gln* | PKDB | P  none | 1 | 30333007 |
| *PKD1* | Exon 39 | truncating | *c.11202C>A* | *p.Tyr3734** | HGMD | P  none | 2 | 26453610 |
| *PKD1* | Exon 40 | truncating | *c.11305_11306delAC* | *p.Thr3769Valfs*46* | novel | P  none | 1 | - |
| *PKD1* | Exon 40 | truncating | *c.11326_11329delTTCA* | *p.Phe3776Alafs*49* | novel | P  none | 3 | - |
| *PKD1* | Exon 41 | missense | *c.11526G>C* | *p.Trp3842Cys* | novel | LP  none | 1 | - |
| *PKD1* | Exon 42 | truncating | *c.11702_11711dupTGCTCACCTC* | *p.Val3905Alafs*59* | novel | P  none | 1 | - |
| *PKD1* | Exon 43 | truncating | *c.11935C>T* | *p.Gln3979** | HGMD/PKDB | P  none | 1 | 21115670  25525159 |
| *PKD1* | Exon 43 | in-frame deletion | *c.11962_11976del* | *p.Arg3988_Ala3992del* | novel | VUS  none | 1 | - |
| *PKD1* | Exon 44 | truncating | *c.12120C>G* | *p.Tyr4040** | novel | P  none | 1 | - |
| *PKD1* | Exon 45 | truncating | *c.12313dupA* | *p.Leu4105Asnfs*52* | HGMD | P  none | 1 | 11967008 |
| *PKD1* | Exon 46 | truncating | *c.12691C>T* | *p.Gln4231** | HGMD/PKDB | P  none | 1 | 19686598 |
| *PKD1* | Exon 46 | truncating | *c.12712C>T* | *p.Gln4238** | HGMD | P  none | 1 | 24611717  30333007 |
| *PKD1* | Exon 46 | truncating | *c.12719delA* | *p.Glu4240Glyfs*118* | novel | P  none | 1 | - |
| *PKD1* | Exon 46 | missense | *c.12826C>T* | *p.Arg4276Trp* | HGMD | VUS  0.43% | 1 | 10200984  20981092 |
| *PKD1* | Intron 25 | splice site | *c.9202-10_9202-3delGTCCTCAC* |  | novel | VUS  none | 2 | - |
| *PKD1* | Intron 37 | splice site | *c.11017-10C>A* | *p.Arg3672fs1** | HGMD/PKDB | P  none | 2 | 10923038  29633482 |
| *PKD1* | Intron 38 | splice site | *c.11156+1G>C* |  | novel | P  none | 3 | - |
| *PKD1* | Intron 5 | splice site | *c.1202-9G>A* |  | HGMD | P  None | 3 | 25491204  24611717 |
| *PKD1* | Intron 8 | splice site | *c.1723-1G>C* |  | novel | P  none | 4 | - |
| *PKD1* |  | deletion 5`UTR |  |  | novel | LP  none | 1 |  |
| *PKD1* |  | deletion Exon 2-11 |  | *p.Leu72fs*23* | HGMD | P  none | 2 | 31027891 |
| *PKD2* |  | deletion Exon1 |  |  | novel | P  none | 1 | - |
| *PKD2* | Exon 1 | truncating | *c.203dupC* | *p.Ala69Glyfs*23* | HGMD | P  none | 1 | 10411676  29633482 |
| *PKD2* | Exon 4 | missense | *c.965G>A* | *p.Arg322Gln* | HGMD | P  0.0009% | 1 | 15772804  22863349 |
| *PKD2* | Exon 5 | missense | *c.1242G>T* | *p.Trp414Cys* | HGMD | LP  none | 1 | 26150605 |
| *PKD2* | Exon 6 | truncating | *c.1340_1346dupCAACAGG* | *p.Gly450Asnfs*23* | HGMD | P  none | 1 | 11968093 |
| *PKD2* | Exon 8 | truncating | *c.1737_1747delinsAACAGGA* | *p.Phe579Leufs*4* | HGMD | P  none | 2 | 31027891 |
| *PKD2* |  | deletion Exon 1-5 |  |  | novel | P  none | 2 | - |
| *PKD2* | Intron 4 | splice site | *c.1094+1G>A* |  | HGMD/PKDB | P  none | 2 | 17100995  25525159 |
| *PKD2* | Intron 9 | splice site | *c.2019+1_20195delGTATG* |  | HGMD | P  none | 1 | 23300259 |
| *PKHD1*  *PKHD1* | Exon 32  Exon 58 | missense  missense | *c.4870C>T*  *c.9370C>T* | *p.Arg1624Trp*  *p.His3124Tyr* | HGMD  HGMD | P  0.022%  P  none | 1 | [11898128](https://www.ncbi.nlm.nih.gov/pubmed/11898128)  12874454 |
| *ALG9* | Exon 4 | truncating | *c.427C>T* | *p.Arg143** | PMID | P  0.0016% | 1 | 31395617 |
| *FLCN* | Exon 13 | missense | *c.1523A>G* | *p.Lys508Arg* | HGMD | LP  0.041% | 1 | 18234728  21538689 |

ACMG: The American College of Medical Genetics and Genomics; *ALG9:* *Alpha-1,2-mannosyltransferase* (NM_001077690.1); *FLCN*: *Folliculin* (NM_144997.6); HGMD: Human Gene Mutation Database (Version 2019.4); No: number of patients with this variant in the analyzed cohort; none: no entry in gnomAD database; *PKD1*: *Polycystic Kidney Disease 1* (NM_001009944.2); *PKD2*: *Polycystic Kidney Disease 2* (NM_000297.3); *PKHD1*: *Polycystic Kidney and Hepatic Disease 1* (NM_138694.3); VUS: variants of unknown significance; PKDB: Polycystic Kidney Disease Database (https://pkdb.mayo.edu/). Variants are listed in order of c.-position. Given gnomAD allele frequencies (gnomAD_AF_) refer to the corresponding ancestry of the index patient, in these cases European Non-Finnish (<https://gnomad.broadinstitute.org/>). Nine formally non-diagnostic, but likely causative *PKD1*-VUS are indicated in **red** (see 9 families with *PKD1*-VUS in Figures 1 and 2A).

**TABLE S3 – 10 ADPKD patients with multiple *PKD1* variants (ACMG class 3-5)**

| ID | Sex /  FHx | Age  [y] | eGFR  [ml/min  /1.73m ^2^] | Age at  ESRD  [y] | Gene | Variant (c.) | Variant (p.) | ACMG  gnomAD_AF_ | Ref | Extrarenal  phenotype |
| --- | --- | --- | --- | --- | --- | --- | --- | --- | --- | --- |
| 4.1 | f /  pos | 57 | 38 | - | ***PKD1***  ***PKD1*** | *c.9898G>A*  *c.4631T>G* | *p.Gly3300Arg*  *p.Val1544Gly* | LP  0.02%  VUS  none | HGMD  novel | PCLD  splenic cysts  pancreatic cysts |
| 12.1 | f /  pos | 66 | <10 | 58 | ***PKD1***  ***PKD1*** | *c.10560dupG*  *c.2965G>A* | *p.Pro3521Alafs**  *p.Ala989Thr* | P  none  VUS  0.0017% | novel  novel | PCLD (LTx)  arach. cyst, T1 DM  diverticulosis  pancreatic cysts |
| 12.4 | f /  pos | 57 | <10 | 41 | ***PKD1***  ***PKD1*** | *c.10560dupG*    *c.2965G>A* | *p.Pro3521Alafs**  *p.Ala989Thr* | P  none  VUS  0.0017% | novel  novel | PCLD (LTx)  KTx  ovarian cysts  HPT |
| 25.1 | f /  n.d. | 62 | <10 | 60 | ***PKD1***  ***PKD1*** | *c.7861G>T*  *c.12826C>T* | *p.Glu2621**  *p.Arg4276Trp* | P  none  VUS  0.43% | novel  HGMD | PCLD  KTx |
| 31.1 | m /  n.d. | 51 | <10 | 39 | ***PKD1***  ***PKD1***  ***PKD1*** | *c.11305_11306delAC*    *c.8611G>A*  *c.9499A>T* | *p.Thr3769Valfs**  *p.Ala2871Thr*  *p.Ile3167Phe* | P  none  VUS  0.01%  VUS  0.21% | novel  novel  HGMD | PCLD  diverticulosis  HPT |
| 33.1 | f /  pos | 77 | <10 | 65 | ***PKD1***  ***PKD1*** | *c.11156G>A*  *c.5953G>A* | *p.Arg3719Gln*  *p.Ala1985Thr* | P  none  VUS  none | PKDB  novel | PCLD  diverticulosis |
| 37.1 | m /  pos | 27 | >90 | - | ***PKD1***  ***PKD1*** | *c.11202C>A*  *c. 10820A>T* | *p.Tyr3734**  *p.Lys3607Met* | P  0.0008%  VUS  none | HGMD  novel | - |
| 37.2 | f /  pos | 48 | <10 | 44 | ***PKD1***  ***PKD1*** | *c.11202C>A*  *c. 10820A>T* | *p.Tyr3734**  *p.Lys3607Met* | P  0.0008%  VUS  none | HGMD  novel | T1 DM  HPT |
| 61.1 | f /  pos | 45 | 67 | - | ***PKD1***  ***PKD1*** | *c.1347_1357*  *delinsACC*  *c.5953G>A* | *p.Ser449Argfs**  *p.Ala1985Thr* | P  none  VUS  none | novel  novel | PCLD |
| 109.2 | m /  pos | 1 | >90 | - | ***PKD1***  ***PKD1*** | *c.974A>G*  *c.8293C>T* | *p.Tyr325Cys*  *p.Arg2765Cys* | LP  none  VUS  0.86% | HGMD  HGMD | congenital hypertension |

ACMG: The American College of Medical Genetics and Genomics; arach: arachnoid; eGFR: estimated glomerular filtration rate (CKD-EPI; ml/min/1.73m^2^); T1 DM: type 1 diabetes mellitus; ESRD: end stage renal disease; f: female; HGMD: Human Gene Mutation Database (Version 2019.4); HPT: hypothyroidism; KTx: kidney transplantation; LP: likely pathogenic; LTx: liver transplantation;none: no entry in gnomAD database; m: male; n. d.: no data; neg: negative; pancrea.: pancreatic; P: pathogenic; PCLD: polycystic liver disease; *PKD1*: *Polycystic Kidney Disease 1* (NM_001009944.2); PKDB, polycystic database Mayo Clinic (https://pkdb.mayo.edu/); pos: positive; VUS: variants of unknown significance. Given gnomAD allele frequencies (gnomAD_AF_) refer to the corresponding ancestry of the index patient, in these cases European Non-Finnish (https://gnomad.broadinstitute.org/).

**TABLE S4 – Additional variants of “*PKD1*/*2*-negative” patients identified by ES**

| ID | Sex /  FHx | Age  [y] | eGFR  [ml/min/1.73m^2^] | Age at  ESRD  [y] | *Gene* | *Variant(c.)/ Zygosity* | *Variant*  *(p.)* | ACMG | Ref. | gnomAD_AF_ | Phenotype |
| --- | --- | --- | --- | --- | --- | --- | --- | --- | --- | --- | --- |
| 20.1 | m /  neg | 70 | <10 | 64 | ***GLI2***  ***SEC63****  ***UGGT1***  ***GPR137B***  ***GPR137B*** | *c.1600G>A (het.)*  *c.1936-8->TTT (het.)*  *c.2654A>G (het.)*  *c.349C>T (het.)*  *c.1091+2->AAGA (het.)* | *p.Val534Met*  *-*  *p.Lys885Arg*  *p.Leu117Phe*  *-* | LP  VUS  VUS  VUS  B | novel  novel  novel  novel  novel | none  0.1%  none  0.068%  0.77% | no liver cysts |
| 35.1 | m /  pos. | 64 | 41 | - | ***TSC2***  ***SEC63**** | *c.2140C>T (het.)*  *c.1936-8->TTT (het.)* | *p.Pro714Ser*  *-* | VUS  VUS | novel  novel | none  0.1% | pancreatic cysts |
| 52.1 | f /  neg | 66 | <10 | 56 | ***COL5A1***  ***COL5A1***  ***GANAB****  ***LRP2***  ***PARN****  ***PKD1*** | *c.2588A>T (het.)*  *c.3418G>A (het.)*  *c.2319G>A (het.)*  *c.6473A>C (het.)*  *c.1405+3A>G (het.)*  *c.971G>T (het.)* | *p.Glu863Val*  *p.Val1140Met*  *p.Ala773Ala*  *p.Asn2158Thr*  *-*  *p.Arg324Leu* | VUS  VUS  VUS  VUS  VUS  LB | HGMD  HGMD  novel  novel  novel  HGMD | 0.04%  0.039%  0.016%  0.061%  0.004%  0.29% | PCLD  HPT  ovarian cysts |
| 77.1 | m /  pos | 77 | 41 | - | ***ALG2***  ***COL4A1***  ***PKHD1***  ***ROBO2***  ***SDCCAG8***  ***TNXB*** | *c.389G>A (het.)*  *c.1454C>T (het.)*  *c.8581A>G (het.)*  *c.3857G>T (het.)*  *c.278C>T (het.)*  *c.2279T>G (het.)* | *p.Arg130Gln*  *p.Pro485Leu*  *p.Ser2861Gly*  *p.Arg1286Leu*  *p.Pro93Leu*  *p.Leu760Trp* | VUS  VUS  LB  VUS  VUS  VUS | novel  novel  HGMD  novel  novel  novel | 0.007%  0.005%  0.37%  0.048%  0.098%  0.0013% | n.d. |
| 97.1 | f /  pos | 68 | <10 | 66 | ***ALG6***  ***ALG6***  ***SEC63****  ***TSC2*** | *c.1442A>G (het.)*  *c.1465T>G (het.)*  *c.1936-8->TTT (pos.hom.)*  *p.275A>T (het.)* | *p.Asn481Ser*  *p.Phe489Val*  *-*  *p.Glu92Val* | VUS  VUS  VUS  VUS | novel  novel  novel  HGMD | 0.014%  0.014%  0.1%  0.1% | PCLD Splenic cysts Diverticulosis |
| 101.1 | m /  pos | 76 | <10 | 70 | ***NOS3***  ***NOTCH3***  ***VEGFB*** | *c.2642C>T (het.)*  *c.4762A>C (het.)*  *c.559C>T (het.)* | *p.Ala881Val*  *p.Asn1588His*  *p.Arg187** | VUS  VUS  VUS | novel  novel  novel | 0.013%  none  0.002% | PCLD Splenic cysts diverticulosis arach. cysts |
| 108.1 | f /  pos | 22 | >90 | - | ***LRP5***  ***CELSR2***  ***GLIS2***  ***SHROOM3***  ***SLIT2*** | *c.1555T>G (het.)*  *c.4508C>A (het.)*  *c.706C>T (het.)*  *c.5027T>A (het.)*  *c.934A>G (het.)* | *p.Trp519Gly*  *p.Ser1503Tyr*  *p.Arg236Cys*  *p.Val1676Asp*  *p.Ile312Val* | LP  LP  LP  VUS  VUS | novel  novel  novel  novel  novel | none  0.046%  0.0016%  none  0.075% | no liver cysts |

ACMG: The American College of Medical Genetics and Genomics; *ALG2: ALG2 alpha:1,3/1,6:mannosyltransferase* (NM_033087.3); *ALG6: ALG6 alpha:1,3:glucosyltransferase* (NM_013339.3); arach: arachnoid; *CELSR2: cadherin EGF LAG seven:pass G:type receptor 2* (NM_001408.2); *COL4A1*: *collagen type IV alpha 1 chain* (NM_001845.5); *COL5A1: collagen type V alpha 1 chain* (NM_000093.4); eGFR: estimated glomerular filtration rate; ESRD: end stage renal disease; f: female; FHx: family history; *GANAB*: *glucosidase II* *alpha subunit* (NM_198335.3); *GLI2 : GLI family zinc finger 2* (NM_005270.4); *GLIS2: GLIS family zinc finger 2* (NM_032575.2); *GPR137B: G protein-coupled receptor 137B* (NM_003272.3); HPT: hypothyroidism; LP: likely pathogenic; m: male; neg: negative; n. d.: no data; *LRP2*: *Low-Density Lipoprotein Receptor-Related Protein 2* (NM_004525.2); *LRP5*: *Low-Density Lipoprotein Receptor-Related Protein 5* (NM_002335.2); none: no entry in gnomAD database; *NOS3: nitric oxide synthase 3* (NM_000603.4); *NOTCH3: notch receptor 3* (NM_000435.2); P: pathogenic; *PARN*: *poly(A)-specific ribonuclease* (NM_002582.3); PCLD: polycystic liver disease; pos: positive; *PKD1: polycystin 1, transient receptor potential channel interacting* (NM_001009944.2); *PKHD1: PKHD1 ciliary IPT domain containing fibrocystin/polyductin* (NM_138694.3); *ROBO2*: *roundabout guidance receptor 2* (NM_002942.4*); SDCCAG8: serologically defined colon cancer antigen 8* (NM_006642.3); *SEC63: SEC63 homolog, protein translocation regulator* (NM_007214.5); *SHROOM3*: *shroom family member 3* (NM_020859.3); SS: splice site; *SLIT2: slit guidance ligand 2* (NM_004787.2); *TNXB*: *tenascin XB* (NM_019105.6); *TSC2: TSC complex subunit 2* (NM_000548.3); *UGGT1: UDP-glucose glycoprotein glucosyltransferase 1* (NM_020120.3); *VEGFB: vascular endothelial growth factor B* (NM_001243733.1); VUS: variant of unknown significance; * - no splice site alteration detected by analysis of patients’ cDNA (Fig. S2). Of note, for ID 101.1; no class 1-3 variant in any known disease gene was detected. Most promising candidate gene variants are highlighted in **red**. Given gnomAD allele frequencies (gnomAD_AF_) refer to the corresponding ancestry of the index patient, in these cases European Non-Finnish (https://gnomad.broadinstitute.org/)

**
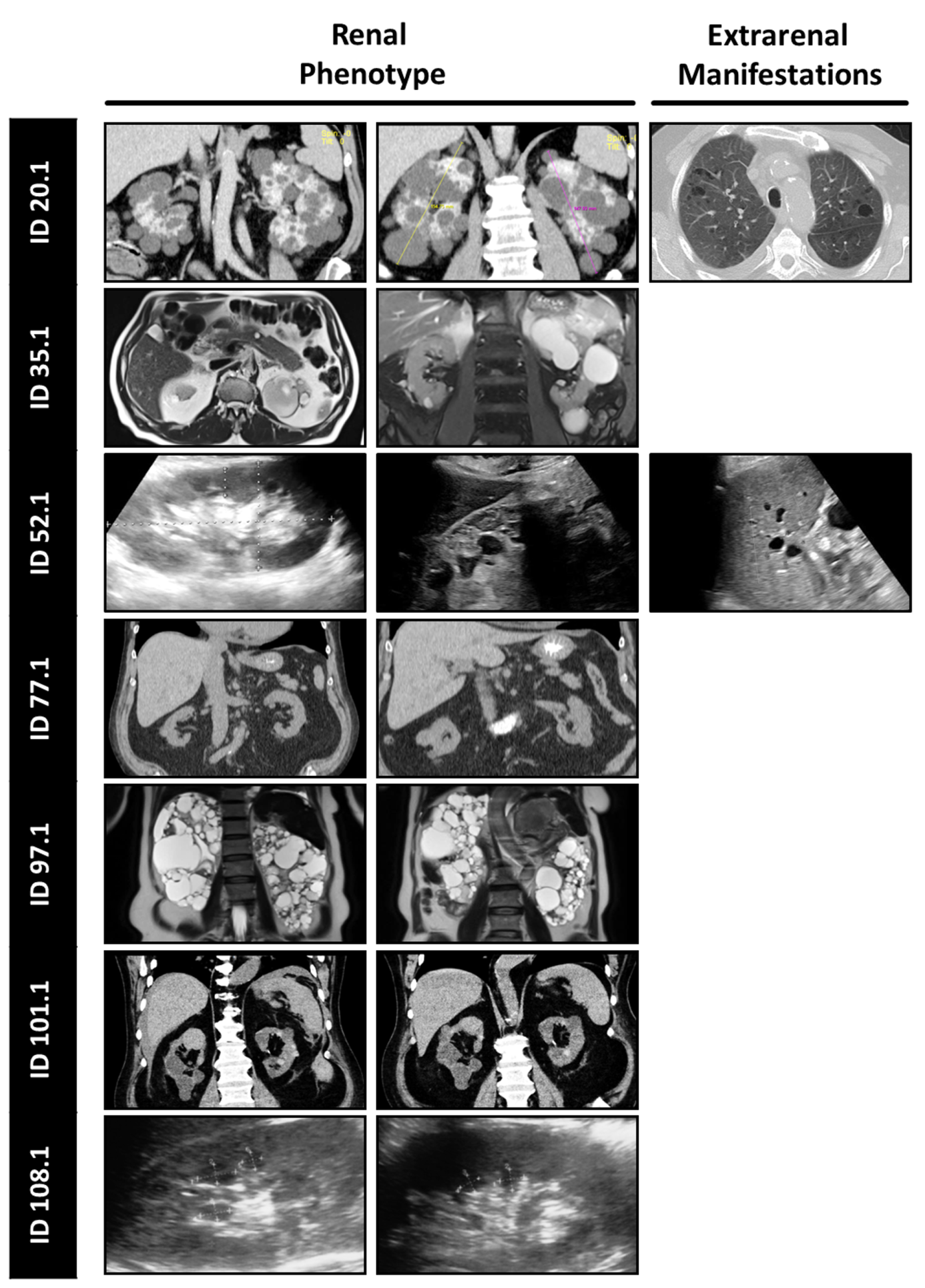
**

**FIGURE S1 – Imaging of bilateral polycystic kidneys in “*PKD1*/*2*-negative” patients**

Renal phenotype: Computed tomography (CT) (ID 20.1; 77.1; 101.1), ultrasound (ID 52.1; 108.1) and magnetic resonance imaging MRI (ID 35.1; 97.1) illustrating bilateral polycystic kidneys. Extra-renal manifestations: CT (ID 20.1) image showing multiple bilateral pulmonary cysts and ultrasound (ID 52.1) showing several small hepatic cystic lesions.

**FIGURE S2 – Illustration of candidate gene findings in GLI2, ALG6, and LRP5**

Two- and three-dimensional illustration of candidate genes variants in: **(A) *GLI2****: GLI family zinc finger 2* (NM_005270.4) c.1600G>A (het.), p.Val534Met within conserved DNA-binding zinc finger domain (**Table S4**) **(B)** ***ALG6*** *alpha:1,3:glucosyltransferase* (NM_013339.3) c.1442A>G (het.), p.Asn481Ser / c.1465T>G (het.), p.Phe489Val within last ER-transmembrane domain (**Table S4**); and **(C) *LRP5***: *Low-Density Lipoprotein Receptor-Related Protein 5* (NM_002335.2) c.1555T>G (het.), p.Trp519Gly within 2^nd^ WD40 repeat (**Table S4**). Upper panel: Partial sequence alignment across different species showing residue Trp519 (W519, red arrow) within an evolutionary highly conserved region of LRP5. Middle panel: Partial sequence alignment of human LRP5 and human LRP6 demonstrating extensive concordance of the 2^nd^ WD40 domain between both members of the low-density lipoprotein receptor (LDLR) family. Residues of identified Trp-changes are denoted by red (p.Trp519 – this study) and blue (p.Trp560 – previous study^19^) arrows, respectively. Lower panel**:** Three-dimensional model, derived from LRP6-crystal structure (3S94^40^), showing a WD40-propeller with Trp-changes in neighboring repeats, indicated by red (Trp519) and blue residues (Trp560). **I** – propeller side view, **II** – propeller top view, **III** – zoomed in view of residues 491 – 571.

**
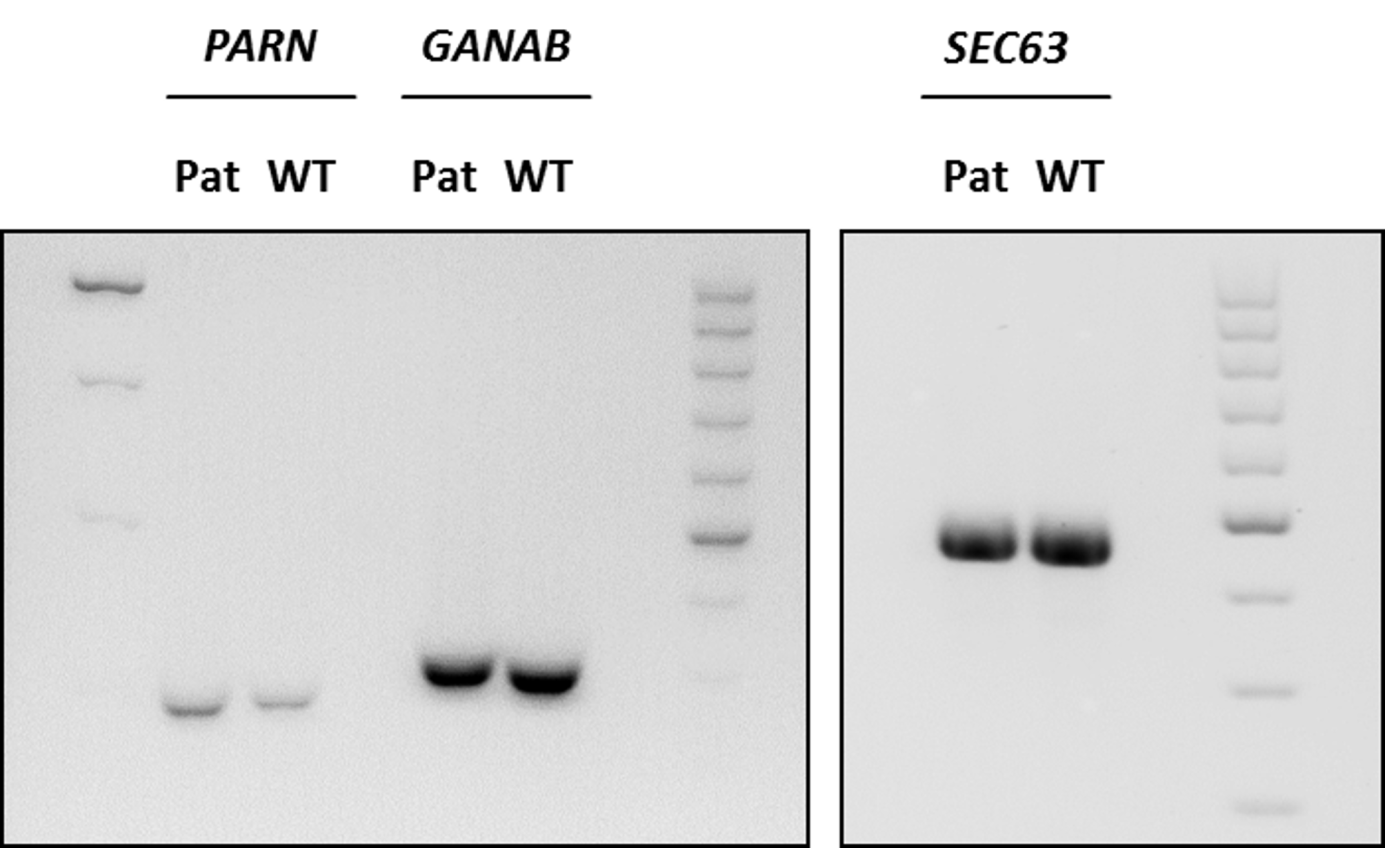
**

**FIGURE S3 – cDNA-based splice site analysis of three variants identified in ES**

Upon RNA-extraction from patients` and control whole blood samples we performed RT-PCR to obtain patient and control cDNA. Next, cDNA based splice site analysis was done by specific primers targeting the following positions: *PARN* (NM_002582.3): c.1405+3A>G and *GANAB* (NM_198335.3): c.2319G>A, p.Ala773= (ID 52.1) (**Table S2**); *SEC63* (NM_007214.4): c.1936-8->TTT (ID 20.1 and ID 97.1) (**Table S2**). After PCR, gel electrophoresis shows equal bands for Wildtype (WT) and Patient (Pat) respectively, indicating no splice site alteration. For verification, all visible bands were extracted and analyzed by Sanger-sequencing, showing no differences between Pat and WT-sequences. All primer sequences are available upon request.
